# Histopathologic Characterization of Experimental Peracute SARS-CoV-2 Infection in the Syrian Hamster

**DOI:** 10.3390/vetsci10090536

**Published:** 2023-08-23

**Authors:** Chad S. Clancy, Kimberly Meade-White, Carl Shaia, Greg Saturday, Heinz Feldmann, Kyle Rosenke

**Affiliations:** 1 Rocky Mountain Veterinary Branch, Division of Intramural Research, National Institutes of Allergy and Infectious Disease, National Institutes of Health, Hamilton, MT 59840, USA; 2 Laboratory of Virology, Division of Intramural Research, National Institutes of Allergy and Infectious Disease, National Institutes of Health, Hamilton, MT 59840, USA

**Keywords:** histopathology, immunohistochemistry, disease modeling, SARS-CoV-2, hamster

## Abstract

**Simple Summary:**

Understanding how a virus interacts with cellular targets in an animal model provides critical information in developing therapeutic targets and vaccines for humans. The Syrian hamster model of SARS-CoV-2 infection, the causative agent of COVID-19, is one of the most widely used animal models for this disease. While extensive work on vaccine and therapeutic interventions have been performed in this animal model, the initial viral targets and spread throughout the respiratory tract has been poorly documented. We sought to evaluate the viral spread by microscopic examination of tissues as early as 6 hours post intranasal inoculation through the development of lung lesions at 3 days post inoculation.

**Abstract:**

Coronavirus Infectious Disease 2019 (COVID-19) initiated a global pandemic that thus far has resulted in the death of over 6.5 million people internationally. Understanding the viral tropism during the initial, subclinical phase of infection is critical to develop targeted vaccines and therapeutics. With the continued emergence of variants of concern, particularly those that appear to have a tropism for the upper respiratory tract, understanding the complete pathogenesis is critical to develop more effective interventions. Thus far, the Syrian hamster has served as the most consistent small animal model of SARS-CoV-2 infection for mild to moderate respiratory disease. Herein, we utilize histopathology and immunohistochemistry to characterize the peracute phase of disease initiating at 6-h-post-inoculation in the intranasal inoculation route Syrian hamster model. Inflammation and viral replication initiates in the respiratory epithelium of nasal turbinates as early as 12-h-post-inoculation and moves caudally through the nasal cavity by 36-h-post inoculation. Lower respiratory involvement can be detected as early as 12-h-post inoculation in the intranasal inoculated hamster model. These data highlight the importance of rostral nasal cavity sampling at early timepoints for detection of SARS-CoV-2 in the Syrian hamster model.

## 1. Introduction

A severe, contagious, pneumonic disease emerged from Wuhan, China on 19 December 2019. A novel betacoronavirus, SARS-CoV-2, was rapidly recognized as the source of respiratory disease [1]. Since the initiation of the pandemic, several animal models of SARS-CoV-2 infection have been developed. Similar to SARS-CoV, the human ACE-2 receptor was rapidly recognized as the cellular entry receptor [2,3]. A humanized ACE-2 receptor knock-in mouse model exists that has shown susceptibility to SARS-CoV-2 infection [4]. However, the K18 mouse model is fatal in part due to neuroinvasion and inflammation associated with aberrant K18 expression within the brain [5]. A non-lethal, mouse adapted SARS-CoV-2 isolate was created circumnavigating the aberrant neural expression of the human ACE2 receptor [6]. However, this model lacks a severe pneumonic disease phenotype with rapid viral clearance by four days post inoculation [6]. More recently, wild type mice have been shown to be susceptible to emerging SARS-CoV-2 variants of concern, though experimentally inoculated wild type mice lack moderate to severe pulmonary histopathology and do not exhibit clinical disease [7,8].

Nonhuman primate models of SARS-CoV-2 infection initiated in the rhesus macaque evaluating two timepoints by necropsy: three- and 21-days post inoculation [9]. This was followed by an additional timepoint of seven days post inoculation within the context of a therapeutic intervention, remdesivir [10]. Additional nonhuman primate models of SARS-CoV-2 infection rapidly emerged evaluating gross and histopathologic changes in the respiratory tract in the cynomolgus macaque [11] and African green monkey [12]. While nonhuman primates continue to be a crucial species for evaluation of vaccines and therapeutics, there are still large knowledge gaps in the pathophysiology of SARS-CoV-2 infection in these species. Due to the high demand for nonhuman primates for pre-clinical evaluation as well as ethical considerations, neither a true natural history study nor minimal infectious dose studies have been conducted in any nonhuman primate species to date.

A well-studied and widely utilized model of respiratory disease in SARS-CoV-2 infection is the Syrian hamster. The Syrian hamster ACE2 receptor naturally supports SARS-CoV-2 interaction and facilitates viral cellular entry [13]. To date, experimental infection models in hamsters have limited the evaluation of the peracute phase of disease to 24 h post-inoculation [14] with most initial work performed at 48 h post inoculation (HPI) [13,15], failing to evaluate the earliest viral tropism and potential lesion formation. Yet, in all cases, a full natural history study evaluating peracute through acute phase of infection in a singular experimental inoculation is lacking. Herein we describe the viral tropism through immunohistochemical evaluation and histologic changes associated with experimental SARS-CoV-2 intranasal inoculation at 6-, 12-, 36-, 48- and 72-HPI in the intranasal inoculated, Syrian hamster model.

## 2. Materials and Methods

### 2.1. Animal Study Approval

Animals were housed and cared for in accordance with standard operating procedures approved by Rocky Mountain Laboratories Institutional Biosafety Committee, Division of Intramural Research, National Institute of Allergy and Infectious Disease, National Institutes of Health. All animal work was approved by the Institutional Animal Care and use Committee and performed in strict accordance with the recommendations described in the guide for the care and use of laboratory animals of the National Institutes of Health, the Office of Animal Welfare, the United States Department of Agriculture in an association for assessment and accreditation of laboratory animal care-accredited facility. Animals were group housed in HEPA-filtered cage systems enriched with nesting material. Commercial food and water were available *ad libitum*. All infectious work with SARS-CoV-2 was performed in the high biocontainment laboratory of the Integrated Research Facility at the Rocky Mountain Laboratories (RML), Division of Intramural Research (DIR), National Institute of Allergy and Infectious Diseases (NIAID), National Institutes of Health (NIH) in accordance with standard operating procedures approved by the Institutional Biosafety Committee (IBC). Samples were inactivated prior to removal from biocontainment according to validated procedures approved by the IBC [16].

### 2.2. Virus

SARS-CoV-2 isolate nCoV-WA1-2020 (MN985325.1) (Vero passage 3) was obtained through CDC and propagated once in Vero E6 cells in DMEM (Sigma, Livonia, MI, USA) supplemented with 2% fetal bovine serum (Gibco, Waltham, MA, USA), 1 mM L-glutamine (Gibco, Waltham, MA, USA), 50 U/mL penicillin and 50 μg/mL streptomycin (Gibco) (virus isolation medium) as previously described [16]. The virus stock was diluted in sterile DMEM (Sigma), without additive, immediately prior to inoculation.

### 2.3. Experimental Design

Thirty-nine Syrian golden hamsters (*Mesocricetus auratus*) 8-weeks-of-age were utilized in the study. Thirty hamsters were inoculated with 10^3^ TCID_50_ WA1 isolate of SARS-CoV-2. Six hamsters, evenly distributed between male and female, were euthanized at each of the predetermined times: 6-, 12-, 36-, 48- and 72-h post inoculation (HPI). In addition, 3 hamsters (male) were mock-inoculated with MEM and 6 hamsters (evenly distributed between male and female) were inoculated with 10^3^ TCID_50_ of gamma irradiated, WA1 SARS-CoV-2. Three gamma-irradiated and three DMEM inoculated hamsters were euthanized at 6 HPI and the remaining 3 gamma-irradiated virus inoculated hamsters were euthanized at 12 HPI. A total inoculum volume of 20 µL divided equally between each naris was utilized for all inoculations. Hamsters were evaluated daily for signs of clinical disease. Euthanasia was performed by bilateral thoracotomy and exsanguination under deep anesthesia. At the time of euthanasia, a mid-sagittal section of the skull, oral section of trachea and all lung lobes were harvested for histopathologic evaluation. A subsection of nasal turbinates, trachea and caudal lung lobes were collected for molecular evaluation.

### 2.4. Histology and Immunohistochemistry

Tissues were fixed in 10% neutral buffered formalin with two changes, for a minimum of 7 days. Tissues were placed in cassettes and processed with a Sakura VIP-6 Tissue Tek, on a 12-h automated schedule, using a graded series of ethanol, xylene, and ParaPlast Extra (Leica, Wetzlar, Germany). Embedded tissues were sectioned at 5 µm and dried overnight at 42 °C prior to staining. Anti-SARS-CoV-2 N-protein immunoreactivity was detected using U864YFA140-4/CB2093 NP-1 (GenScript, Piscataway, NJ, USA) at a 1:1000 dilution. The secondary antibody is the Vector Laboratories ImPress VR anti-rabbit IgG polymer (cat# MP-6401). The tissues were then processed for immunohistochemistry using the Discovery Ultra automated processor (Ventana Medical Systems, Oro Valley, AZ, USA) with a ChromoMap DAB kit (Roche Tissue Diagnostics cat#760-159, Basel, Basel-Stadt, Switzerland). Histopathology slide readouts and immunohistochemical analysis were performed blindly by a board certified veterinary anatomic pathologist. Representative images of each timepoint were captured.

### 2.5. Virology

qPCR was performed on RNA extracted from swabs collected immediately prior to euthanasia using QiaAmp Viral RNA mini kit (Qiagen, Germantown, MD, USA) according to the manufacturer’s instructions. Tissues (30 mg or less) were homogenized in RLT buffer and RNA was extracted using the RNeasy kit (Qiagen, Germantown, MD, USA) according to the manufacturer protocol. Viral RNA was detected with a one-step real-time RT-PCR assay (Quantifast, Qiagen, Germantown, MD, USA) using primers and probes generated to amplify the E gene and with custom primers designed against the SARS-CoV-2 E protein as previously described [16,17].

## 3. Results

### 3.1. Gross Pathology

Clinical signs of disease were not observed during the course of the study. No grossly observable lesions were observed in either the upper or lower respiratory tract at 6-HPI in experimentally inoculated hamsters, gamma-irradiated virus inoculated and mock inoculated negative control hamsters. Early evidence of broncho-interstitial pneumonia was grossly observed as early as 12-HPI and were characterized by well-demarcated, variably sized, red foci disseminated throughout the cranial portions of both left and right lung lobes in experimentally inoculated hamsters. Grossly observable pulmonary changes persisted through 72-HPI.

### 3.2. Histopathology

#### 3.2.1. Six Hours Post Inoculation

**Figure 1:**
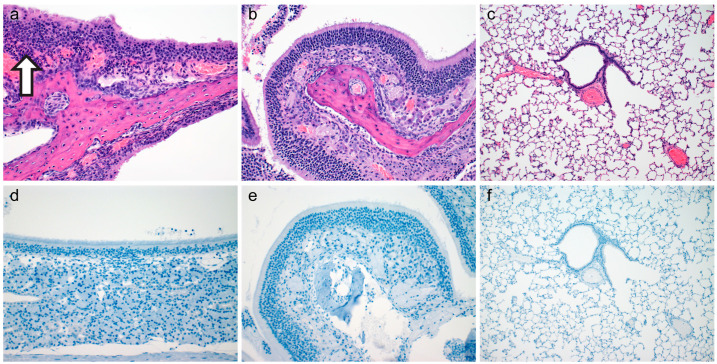
Histopathology and Immunohistochemistry at Six Hours Post Inoculation. Histopathology of ciliated respiratory epithelium ((**a**), open arrow highlighting moderate neutrophilic infiltrate, 200×), olfactory epithelium ((**b**), 200×), and pulmonary tissue ((**c**), 100×) at six-hours-post-inoculation. Immunohistochemical detection of SARS-CoV-2 N protein in ciliated respiratory epithelium ((**d**), 200×), olfactory epithelium ((**e**), 200×), and pulmonary tissue ((**f**), 100×) at six-hours-post-inoculation.

Post-inoculation inflammation was detected in the respiratory ciliated epithelium of the nasal turbinates as soon as 6-HPI in 10^3^ SARS-CoV-2 WA-1 isolate inoculated hamsters. Low numbers of neutrophils infiltrated the lamina propria-submucosa and rarely extended into the overlying mucosa (Figure 1a). Rhinitis within the ciliated epithelium was observed in 67% (n = 4/6) evaluated hamsters and ranged from minimal to moderate (Figure 1a). No inflammation was observed in olfactory epithelium (Figure 1b) or within the lower respiratory tree (Figure 1c). Immunohistochemistry of the entire respiratory tree failed to highlight viral antigen at 6-HPI (Figure 1d–f). Neither inflammation nor immunoreactive epithelial cells were detected in gamma-irradiated virus inoculated or media inoculated hamsters at 6-HIP. A summary of lesion presence and immunohistochemical findings are found in Table 1.

#### 3.2.2. Twelve Hours Post Inoculation

**Figure 2:**
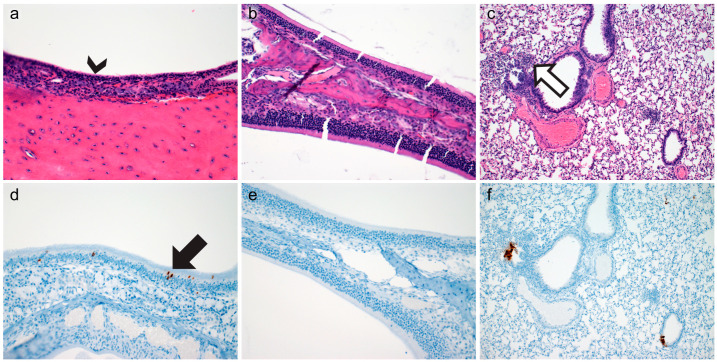
Histopathology and Immunohistochemistry at Twelve Hours Post Inoculation. Histopathology of ciliated respiratory epithelium ((**a**), arrowhead highlighting ciliocytophthoria, 200×), olfactory epithelium ((**b**), 200×), and pulmonary tissue ((**c**), open arrow highlighting mild interstitial pneumonia 100×) at twelve-hours-post-inoculation. Immunohistochemical detection of SARS-CoV-2 N protein in ciliated respiratory epithelium ((**d**), arrow highlighting rare immunoreactive cells, 200×), olfactory epithelium ((**e**), 200×), and pulmonary tissue ((**f**), 100×) at twelve-hours-post-inoculation.

Rhinitis within the ciliated respiratory epithelium was characterized by infiltration of neutrophils within the lamina propria-submucosa and the overlying mucosa accompanied with loss of apical cilia (ciliocytophthoria, Figure 2a, arrowhead). Rhinitis within the ciliated epithelium was observed in 50% (n = 3/6) of evaluated hamsters and ranged from minimal to mild. Evidence of acute inflammation was not observed within the olfactory epithelium (Figure 2b). Lower respiratory tract inflammation was observed in both the conducting airways and pulmonary parenchyma (Figure 2c). Bronchiolitis was primarily characterized by infiltration of the lamina propria by low numbers of neutrophils with rare lymphocytes. Interstitial inflammation was characterized by minimal to mild expansion of alveolar septa by neutrophils and lymphocytes with rare foci of extension into adjacent alveolar spaces. Within the nasal turbinates, SARS-CoV-2 immunoreactivity was observed in the ciliated respiratory epithelium (Figure 2d) but not the olfactory epithelium (Figure 2e). Bronchiolar epithelia were rarely immunoreactive (Figure 2f) along with exceedingly rare type I pneumocytes. Neither inflammation nor immunoreactive epithelial cells were noted in gamma-irradiated inoculated virus hamsters at 12-HPI. A summary of lesion presence and immunohistochemical findings are found in Table 1.

#### 3.2.3. Thirty-Six Hours Post Inoculation

**Figure 3:**
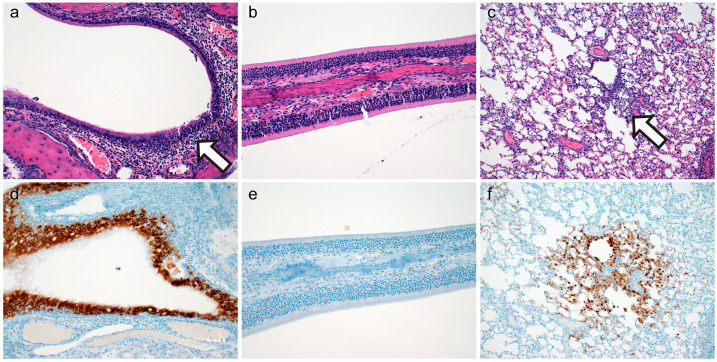
Histopathology and Immunohistochemistry at Thirty-Six Hours Post Inoculation. Histopathology of ciliated respiratory epithelium ((**a**), open arrow highlighting moderate numbers of infiltrating leukocytes within the lamina propria, 200×), olfactory epithelium ((**b**), 200×), and pulmonary tissue ((**c**), arrow highlighting mild interstitial pneumonia, 100×) at thirty-six-hours-post-inoculation. Immunohistochemical detection of SARS-CoV-2 N protein in ciliated respiratory epithelium ((**d**), 200×), olfactory epithelium ((**e**), 200×), and pulmonary tissue ((**f**), 100×) at thirty-six-hours-post-inoculation.

Mild to moderate rhinitis centered on the ciliated respiratory epithelium was observed in 100% (n = 6/6) of evaluated hamsters at 36-HPI. Rhinitis was characterized by moderate numbers of neutrophils and lymphocytes infiltrating and expanding the lamina-propria-submucosa and occasionally extending through the mucosa and into the lumen (Figure 3a). Olfactory epithelium inflammation was not observed at 36-HPI (Figure 3b). Minimal to mild interstitial pneumonia with cellular exudate in alveolar spaces was observed in 33% (n = 2/6) of evaluated hamsters at 36 HPI (Figure 3c). Abundant N-protein antigen was detected by immunohistochemistry within the ciliated respiratory epithelium at 36 HPI (Figure 3d). No immunoreactivity was observed at 36 HPI in olfactory epithelium (Figure 3e). Multifocally throughout lung lobes, bronchioles and adjacent alveolar spaces exhibited immunoreactivity to SARS-CoV-2 N-protein (Figure 3f). A summary of lesion presence and immunohistochemical findings are found in Table 1.

#### 3.2.4. Forty-Eight Hours Post Inoculation

**Figure 4:**
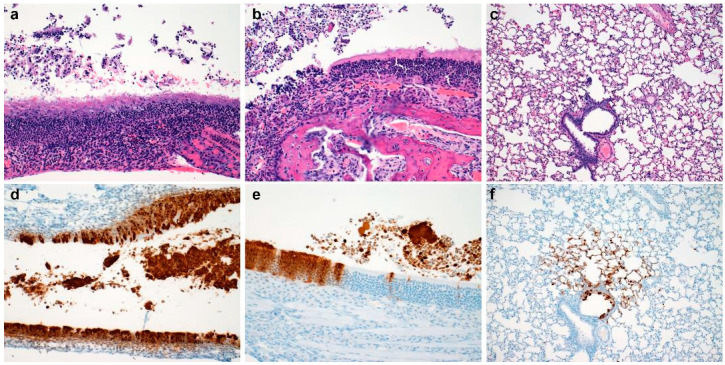
Histopathology and Immunohistochemistry at Forty-Eight Hours Post Inoculation. Histopathology of ciliated respiratory epithelium ((**a**), moderate rhinitis, 200×), olfactory epithelium ((**b**), erosion and mild inflammation, 200×), and pulmonary tissue ((**c**), 100×) at forty-eight-hours-post-inoculation. Immunohistochemical detection of SARS-CoV-2 N protein in ciliated respiratory epithelium ((**d**), 200×), olfactory epithelium ((**e**), 200×), and pulmonary tissue ((**f**), 100×) at forty-eight-hours-post-inoculation.

Mild to severe histopathologic lesions were observed throughout the nasal turbinates in both ciliated respiratory epithelium (Figure 4a) and olfactory epithelium (Figure 4b) at 48-HPI. In regions of respiratory epithelium, the lamina propria-submucosa were frequently infiltrated by mild to moderate numbers of lymphocytes with fewer neutrophils that frequently infiltrated the overlying mucosa and accumulated within the lumen. Occasionally, inflammatory infiltrates extended to adjacent submucosal glands. Within the olfactory epithelium, foci of mild inflammation were characterized by infiltration of low numbers of neutrophils and/or lymphocytes extending to severe inflammation in which the submucosa was entirely denuded of olfactory epithelium and replaced by degenerate leukocytes and cellular debris. Minimal to mild interstitial pneumonia was observed in 83% (n = 5/6, Figure 4c) of experimentally inoculated hamsters at 48-h post-inoculation. Antigen was detected by immunohistochemistry throughout the entire pulmonary tree at 48-HPI (Figure 4d–f). A summary of lesion presence and immunohistochemical findings are found in Table 1.

#### 3.2.5. Seventy-Two Hours Post Inoculation

**Figure 5:**
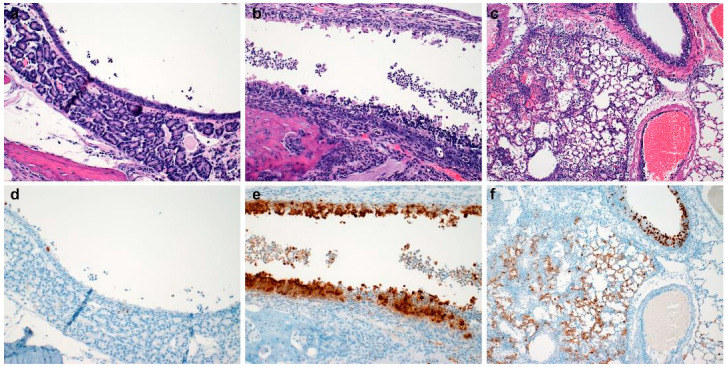
Histopathology and Immunohistochemistry at Seventy-Two Hours Post Inoculation. Histopathology of ciliated respiratory epithelium ((**a**), 200×), olfactory epithelium ((**b**), 200×), and pulmonary tissue ((**c**), 100×) at seventy-two-hours-post-inoculation. Immunohistochemical detection of SARS-CoV-2 N protein in ciliated respiratory epithelium ((**d**), moderate broncho-interstitial pneumonia, 200×), olfactory epithelium ((**e**), 200×), and pulmonary tissue ((**f**), 100×) at seventy-two-hours-post-inoculation.

Moderate to severe histopathologic lesions were observed throughout the nasal turbinated at 72-h-post inoculation, both in ciliated and olfactory epithelium (Figure 5a,b). Histologic lesions in both microanatomic regions consisted of influx of moderate numbers of degenerate and non-degenerate neutrophils in the lamina propria with or without lamina propria expansion by edema fluid. The luminal space frequently contained abundant cellular debris admixed with degenerate and non-degenerate leukocytes. In contrast, pulmonary lesions ranged from minimal to moderate with minimal to mild interstitial pneumonia observed in 83% (n = 5/6) of experimentally inoculated hamsters, minimal to moderate bronchiolitis observed in 100% (n = 6/6) hamsters and minimal to moderate cellular exudate observed in 83% (n = 5/6) of hamsters (Figure 5c). At 72 HPI, SARS-CoV-2 N protein immunoreactivity was observed in every evaluated epithelial type in both the upper respiratory and lower respiratory tree, though immunoreactivity in ciliated respiratory epithelia were rare (Figure 5d–f). A summary of lesion presence and immunohistochemical findings are found in Table 1.

### 3.3. Virology

**Figure 6:**
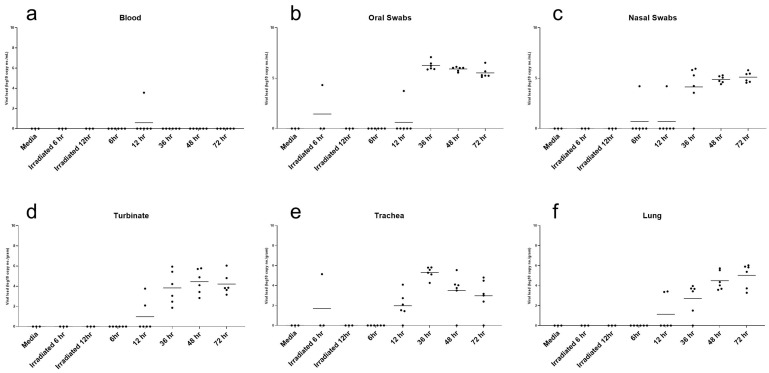
Molecular Detection of Subgenomic E SARS-CoV-2 in Tissues and Swabs. Detection of SARS-CoV-2 RNA by PCR in blood (**a**), oral swabs (**b**), nasal swabs (**c**), nasal turbinates (**d**), trachea (**e**), and lung (**f**) from 6- to 72-h-post-inoculation.

At the time of euthanasia, whole blood, nasal swabs, oral swabs, nasal turbinates, rostral trachea, and lungs were collected for molecular analysis. To elucidate the viral load associated with replicating virus, PCR for subgenomic E protein was performed (Figure 6). A very low level of subgenomic E RNA was only detected in a single blood sample from a 12 HPI inoculated hamster (Figure 6a). Replicating viral RNA was not detected in the blood of media or irradiated virus inoculated hamsters, nor hamsters evaluated at 6, 36, 48 or 72-h post inoculation. Oral and nasal swabs revealed subgenomic E viral RNA in all evaluated hamsters as early as 36-h post inoculation (Figure 6b,c). Viral RNA was detectable in the nasal turbinates of hamsters as early as 12-h post inoculation and remained detectable in all hamsters evaluated at 36, 48 and 72-h-post inoculation (Figure 6d). Similarly, subgenomic E viral RNA loads were observed in the trachea as early as 12-h-post inoculation and remained present throughout the study (Figure 6e). Lastly, subgenomic E viral RNA was detected in two of six lung samples at 12-h-post inoculation with steady increases throughout the study (Figure 6f).

## 4. Discussion

In this study, we sought to define the natural history of the peracute infection of SARS-CoV-2 in the Syrian hamster model using a clinically derived, human isolate (WA-1). In contrast to human infections, clinical signs of respiratory disease were not observed in any hamster at the timepoints evaluated in this model. In this intranasal inoculation model, we show through immunohistochemistry, infection initiates in the upper respiratory tract within respiratory epithelium, not olfactory epithelium. Previous work with the same viral stock in similarly sourced Syrian hamsters within the same facility showed viral antigen in the olfactory epithelium at 24 HPI following intranasal inoculation [14]. In this intranasal inoculation model, we show through immunohistochemistry, infection initiates in the upper respiratory tract within respiratory epithelium, not olfactory epithelium. Turbinate homogenates showed viral replication through subgenomic E PCR analysis as early as 12 HPI, but consistent presence was not observed until 36 HPI. This mimics the rare immunoreactive cells observed in respiratory epithelium at 12 HPI with abundant cellular immunoreactivity observed at 36 HPI. Previous studies have shown mixed immunoreactivity in nasal turbinates at 24 HPI [14]. On molecular analysis, turbinate homogenates utilized for viral culture are incapable of differentiating olfactory or respiratory cell origin, highlighting the utility of imaging analysis such as immunohistochemistry. Oral and nasal swabs are frequently utilized as a non-lethal method of detection for viral replication and shedding in the upper respiratory tree. Both oral and nasal swabs mimicked immunohistochemical results with limited detection at 6 and 12 HPI but consistent viral detection by PCR at 36 through 72 HPI. Consistently, animals that expressed immunoreactivity were also positive by PCR.

Interestingly, conducting airway and lower respiratory tract infection can be observed within conducting airways as early as 12 HPI. This may be an artifact of the model, as the virus is diluted in 20 µL of media and slowly deposited into each naris under general anesthesia, which may overwhelm the conducting airways of the upper respiratory tract and allow some movement into the lower respiratory tree. However, the lack of viral detection by PCR in the lower respiratory tree at 6 HPI with limited detection in the upper respiratory tree at this time suggests that the viral spread is not due to the route of inoculation. This hypothesis is supported by the presence of viral RNA in the oral swabs of WA-1 inoculated but not media inoculated or irradiated virus inoculated hamsters suggesting robust, early viral replication and secretion in fluids that drain toward to oropharynx as early as 6 HPI.

Importantly, rodents have defined gross and microanatomic differences in the upper respiratory tract relative to humans. Rodents have a small vestibule, branched and scrolling ethmoturbinates, transverse lamina with an olfactory recess and lack of nasopharyngeal tonsillar tissue relative to humans and nonhuman primates [18]. Rodents have extensive olfactory epithelium comprising approximately 45–50% of the entire respiratory tissues relative to the 3–5% of total surface area observed in humans and nonhuman primates [19,20]. Acknowledging the differential microanatomic structure in model species is critical to interpretation and clinical application of infectious disease models. In both humans and rodents, the bulk of the non-olfactory epithelium in the upper respiratory tree is populated by respiratory epithelium, the site of earliest viral antigen in our study. Identification of this early viral tropism may better recapitulate the upper respiratory infection observed clinically in humans naturally infected with SARS-CoV-2 and explain how high viral loads are released during upper respiratory infection.

Importantly, this work shows evaluation of respiratory epithelium in the rostral nasal passage rather than the olfactory epithelium in the caudal nasal passage is critical to detect peracute viral replication. The microanatomic difference, with less surface area occupied by respiratory epithelium in the hamster relative to humans, may explain why early viral shedding is not detected rapidly or at a high copy number with exterior nasal swabs in the hamster model. Interestingly, respiratory epithelial inflammation is relatively mild and transient compared to olfactory epithelial inflammation in the hamster model. True denudation of respiratory epithelium was never observed in this peracute study through 72 HPI while olfactory epithelial sloughing and full epithelial erosion was consistently observed at later time points. This intense inflammatory response was accompanied with abundant immunoreactivity for SARS-CoV-2 within olfactory epithelial cells. Interestingly, respiratory epithelium was capable of regeneration and viral clearance while abundant virus remained detectable within the olfactory epithelium.

In infectious disease research, immunohistochemistry is best suited for qualitative imaging analysis to accompany molecular assays as a quantitative analytic tool. In natural history studies, immunohistochemical analysis coupled with histopathologic evaluation is of particular importance as it provides insight into early cell targets and pathways of systemic spread. In the case of SARS-CoV-2 in the intranasally inoculated Syrian hamster model, immunohistochemistry highlights the rapid spread of viral antigen from the respiratory epithelium of the upper respiratory tree, the site of inoculation, to the conducting airways in the lower respiratory tree. Additionally, immunohistochemical evaluation highlights the delayed viral tropism for the abundant olfactory epithelium. This information provides key insight into critical early microanatomic sites of peracute infection, guiding decisions on microanatomic regions that need to be analyzed for evaluation of early phase interventional therapies. Importantly, this work supports the combined use of molecular assays, histologic imaging and immunohistochemical analysis on sampled tissue sets.

Taken together, molecular analysis and immunohistochemical analysis support early viral replication in the respiratory epithelium of the Syrian hamster with limited, virus associated, tissue damage in the infected microanatomic regions. Additionally, immunohistochemistry aids molecular analysis in identifying varying viral tropism during the progression of upper respiratory tract infection associated with SARS-CoV-2 infection.

## Figures and Tables

**Table 1:** Immunohistochemical Detection of N-Protein in Evaluated Anatomic Regions. − is not detected, + is detected, rare is scattered individualized cells.

Immunohistochemical Detection of SARS-CoV-2 N-Protein			
	Ciliated Respiratory Epithelium	Neurosensory Olfactory Epithelium	Lower Respiratory Tree
6 h Post Inoculation	−	−	−
12 h Post Inoculation	Rare	−	Rare
36 h Post Inoculation	+	−	+
48 h Post Inoculation	+	+	+
72 h Post Inoculation	Rare	+	+
Irradiated Virus Control	−	−	−

## Data Availability

All data is presented here. Additional information can be requested through the corresponding authors.
